# Venous Thromboembolism in Asian Patients with Pancreatic Cancer Following Palliative Chemotherapy: Low Incidence but a Negative Prognosticator for Those with Early Onset

**DOI:** 10.3390/cancers10120501

**Published:** 2018-12-10

**Authors:** Jen-Shi Chen, Chia-Yen Hung, Hung Chang, Chien-Ting Liu, Yen-Yang Chen, Chang-Hsien Lu, Pei-Hung Chang, Yu-Shin Hung, Wen-Chi Chou

**Affiliations:** 1 Departments of Hematology-Oncology, Chang Gung Memorial Hospital at Linkou and Chang Gung University College of Medicine, Taoyaun 333, Taiwan; js1101@cgmh.org.tw (J.-S.C.); eliza7301@gmail.com (C.-Y.H.); horng@cgmh.org.tw (H.C.); f22338@cgmh.org.tw (Y.-S.H.); 2 Division of Hematology-Oncology, Department of Internal Medicine, Mackay Memorial Hospital, Taipei 104, Taiwan; 3 Department of Hematology-Oncology, Chang Gung Memorial Hospital at Kaohsiung, Kaohsiung 833, Taiwan; m7155@cgmh.org.tw (C.-T.L.); chenyy@cgmh.org.tw (Y.-Y.C.); 4 Departments of Hematology-Oncology, Chang Gung Memorial Hospital at Chiayi, Chiayi 612, Taiwan; luchanghsien@gmail.com; 5 Department of Hematology-Oncology, Chang Gung Memorial Hospital at Keelung, Keelung 204, Taiwan; ph555chang@cgmh.org.tw

**Keywords:** pancreatic cancer, venous thromboembolism, incidence, predictor, outcome

## Abstract

Background: Few studies have reported the epidemiology and clinical outcome of venous thromboembolism (VTE) in Asian patients with pancreatic cancer. This study investigated the incidence, risk factors, and clinical outcome of VTE in patients with pancreatic cancer following palliative chemotherapy. Methods: The medical records of 838 patients with newly diagnosed locally advanced or metastatic pancreatic cancer who underwent palliative chemotherapy between 2010 and 2016 at four institutes in Taiwan were retrospectively reviewed. The clinical characteristics of all patients were analyzed to identify independent predictors of VTE and their effects on survival outcome. Results: During the median follow-up period of 7.7 months (range, 0.6–55.6), VTE occurred in 67 (8.0%) of the 838 patients. Leukocyte count > 11,000/μL and presence of liver metastases were the independent predictors of VTE. Patients with VTE did not show significantly poorer survival outcomes than those without VTE. However, early-onset VTE that occurred within 1.5 months after chemotherapy initiation was an independent negative prognosticator for overall survival. Conclusion: VTE incidence was found to be lower in Asian patients with pancreatic cancer than in their Western counterparts. Early-onset VTE, but not late-onset VTE, is a negative prognosticator for survival outcomes.

## 1. Introduction

Venous thromboembolism (VTE) is a common complication in patients with cancer. The presumptive pathophysiologic mechanism involves the hypercoagulable state accompanying cancer [1,2,3,4], compression of major veins by tumor direct invasion [5], or precipitation by antitumor treatments including some chemotherapeutic agents [6,7] and surgery [8]. The risk of VTE is 2–7-fold higher in patients with cancer than in those without cancer, and the highest risk is observed in patients with cancer subtypes including pancreatic, liver, gastrointestinal, lung, and hematological cancer [9,10,11]. VTE affects the survival outcomes of patients with cancer; that is, it increases mortality by 2–6-fold, compromising their quality of life [12,13,14,15]. 

Several guidelines from Western countries recommend the primary prophylaxis of anticoagulation for high-risk cancer patients undergoing anticancer treatment [16,17,18]. Two important randomized studies demonstrated the high efficacy and feasibility of prophylactic low-molecular weight heparin, accompanied with 58–60% risk reduction, in prevention of symptomatic VTEs among advanced pancreatic cancer patients undergoing palliative chemotherapy [19,20]. However, these guidelines have never been widely applied to Asian patients with cancer because a lower prevalence of VTE has been reported in Asian patients, and risk factors for VTE in the Asian population have been rarely explored. In general, the incidence of VTE among the Asian general population and Asian patients with cancer is lower than that in the Western population [10,21,22]. In an early study, VTE incidence was found to be 5.3% among Korean advanced pancreatic cancer patients [23], which is lower than that observed in other ethnic groups (15–28%) [19,20]. However, few studies have reported the epidemiology and clinical outcome of VTE in Asian patients with cancer. Furthermore, numerous parameters with predictive value for VTE have been identified in patients with cancer, including cancer subtypes, parameters of blood count, body mass index (BMI) [24], concomitant medication, and chemotherapy agents [25]. Pancreatic cancer is one of the most well-recognized common cancers associated with VTE [26]. Whether risk factors for VTE observed in Western patients with cancer can be generalized to Asian patients with pancreatic cancer remains uncertain. Thus, this study investigated the incidence, risk factors, and clinical outcome of VTE in patients with pancreatic cancer who underwent palliative chemotherapy.

## 2. Result

### 2.1. Patients’ Characteristics

The demographic characteristics of 838 patients are shown in Table 1. The median age was 62 years (range, 23–89), and 59.3% were men. The median BMI was 22.5 kg/m^2^ (range, 13–36.2), and only three patients (0.4%) had a BMI over 35 kg/m^2^. Moreover, 73% had comorbidities, and the most common comorbidities were hypertension (39.6%), diabetes (37.4%), and coronary artery disease (6.2%). Overall, 78.2% had stage IV disease, and the three most common metastatic sites were the liver (52.3%), peritoneum (28.5%), and distant lymph nodes (17.9%). No statistical differences were observed in age, sex, BMI, ECOG PS, Charlson comorbidity index (CCI), tumor site, tumor stage, tumor grade, presence of drainage for jaundice, tumor markers, chemotherapy regimens, and distribution of Khorana risk score between the non-VTE and VTE groups. Presence of liver metastases and leukocyte count > 11 × 10^9^/L in peripheral blood were more predominantly found in patients with VTE than in those without VTE.

### 2.2. Incidence and Predictors of Venous Thromboembolism (VTE)

**Figure 1:**
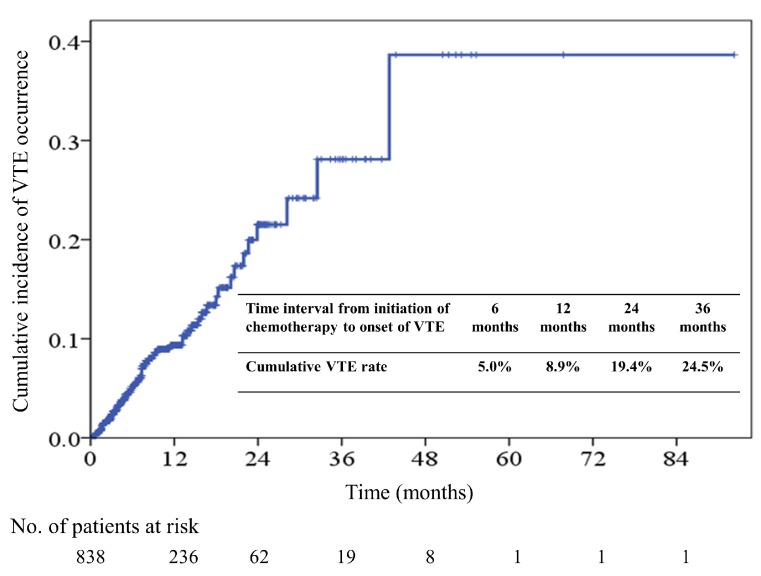
Cumulative incidence of Venous Thromboembolism (VTE) in all patients.

**Figure 2:**
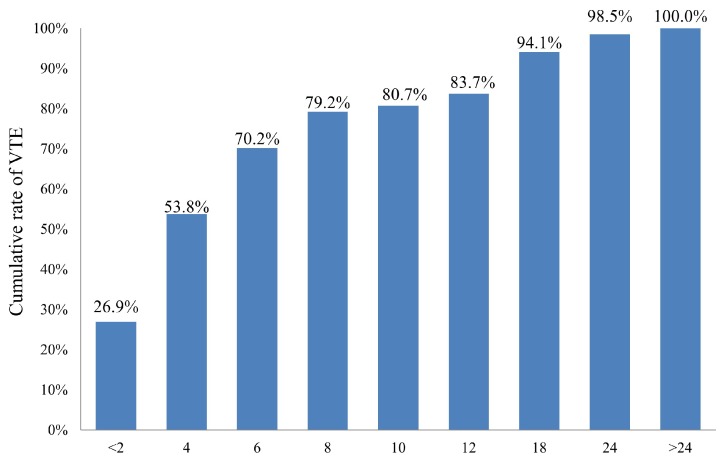
Cumulative rate of VTE among 67 VTE patients.

During the median follow-up period of 7.7 months (range, 0.6–55.6), 754 patients (90.0%) died at the study end. VTE occurred in 67 (8.0%) of 838 patients; the detailed distribution of VTE in our study group is shown in Table 2. Of 67 patients with VTE, 34 (50.7%) had deep vein thrombosis (DVT) of lower limbs, 14 (20.9%) had pulmonary embolism (PE), and 8 (11.9%) had concomitant DVT and PE. The 6-, 12-, 24-, and 36-month cumulative incidence rates of VTE were 5.0%, 8.9%, 19.4%, and 24.5%, respectively (Figure 1). Of 67 patients who developed VTE, 26.9%, 53.8%, and 70.2% of VTE occurred within 2, 4, and 6 months of chemotherapy initiation, respectively. The cumulative incidence of VTE among 67 patients after chemotherapy initiation is shown in Figure 2.

**Figure 3:**
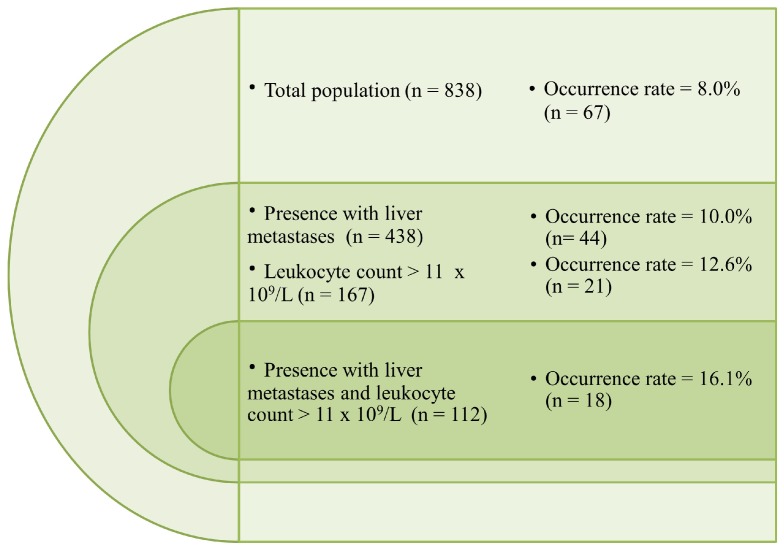
Distribution of VTE according to the risk factors presented by patients.

The results of univariate and multivariate analyses of clinical factors for predicting VTE occurrence are shown in Table 3. Both univariate and multivariate analyses identified leukocyte count > 11,000/μL (12.6% vs. 6.6% of patients who had leukocyte counts > and ≤ 11,000/μL, adjusted hazard ratio [HR] = 1.75, 95% CI: 1.07–3.03; *p* = 0.032) and presence of liver metastases (10.5% vs. 3.7% of patients with and without liver metastases, adjusted HR = 1.65, 95% CI: 1.03–3.99; *p* = 0.046) as independent predictors of VTE. Figure 3 illustrates the distribution of VTE according to the risk factors presented by patients. For patients who presented with liver metastases, leukocyte count > 11,000/μL, and both risk factors, the VTE incidence rate was 10.0% (44/438), 12.6% (21/167), and 16.1% (18/112), respectively.

### 2.3. Association of Khorana Risk Score with VTE Incidence

Patients with a Khorana risk score of 4–5 showed a higher tendency to develop VTE than those with a Khorana risk score of 2–3 (11.2% vs. 7.1%, HR = 1.66, 95% CI: 0.97–2.87; *p* = 0.067). However, no statistically significant difference was observed between the two groups in multivariate analysis. The data were further stratified by the Khorana risk score to clarify whether the Khorana risk score is associated with the VTE incidence rate. The overall VTE incidence rate was 99 per 1,000 person-years. The incidence rate was higher in patients with Khorana risk scores of 4 and 5 than in those with Khorana risk scores of 2 and 3. However, based on nonsignificant differences in HR estimates, patients with a higher Khorana risk score did not have a significantly higher incidence rate than those with a Khorana risk score of 2. Adjustment for leukocyte count and presence of liver metastases did not result in any marked difference in HR estimates (Table 4).

### 2.4. Survival Outcome

**Figure 4:**
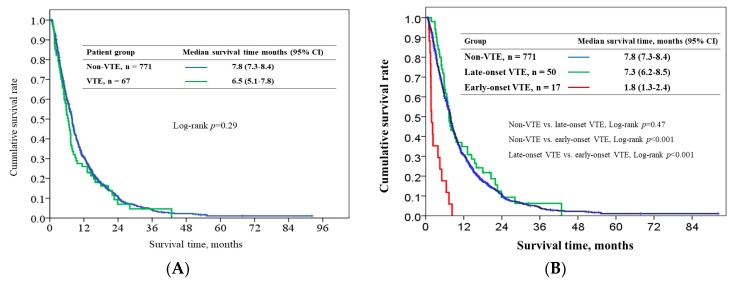
Survival outcome in VTE and non-VTE patients (**A**) and in early-onset, late-onset, and non-VTE patients (**B**).

The overall median survival in the VTE and non-VTE groups were 6.5 (95% CI: 5.1–7.8) and 7.8 months (95% CI: 7.3–8.4). No difference was observed in the survival time of the two groups after chemotherapy initiation (*p* = 0.29) (Figure 4A). Patients with early-onset VTE had poor overall median survival outcomes (1.8 months, 95% CI:1.3–2.4) than those with late-onset VTE (7.3 months, 95% CI:6.2–8.5, *p* < 0.001) and those without VTE (*p* < 0.001). No difference was observed in survival time between the late-onset VTE and non-VTE groups (*p* = 0.47) (Figure 4B). Table 5 presents the results of univariate and multivariate analyses of clinical factors for predicting overall survival (OS). In univariate analysis, patients with VTE did not show poorer survival outcomes than those without VTE. Early-onset VTE was a poor prognostic variable in both univariate and multivariate analyses (adjusted HR = 3.80, 95% CI: 2.34–6.18; *p* < 0.001).

## 3. Discussion

To the best of our knowledge, this is the largest study to evaluate the incidence, risk factors, and clinical outcome of VTE in Asian patients with pancreatic cancer following palliative chemotherapy. We found that VTE occurred in 8.0% of 838 patients with advanced pancreatic cancer, with an incidence rate of 99 per 1000 person-years. Our study identified that presence of liver metastases and leukocyte count > 11,000/μL were the two most accurate predictors of VTE. Patients with VTE did not show significantly poorer survival outcomes than those without VTE. However, VTE occurrence within 1.5 months after chemotherapy initiation was an independent poor prognostic factor for OS. We believe that the results of this retrospective real-world analysis of patients from four institutes in Taiwan evaluated over a 7-year period are informative and provide the crude incidence rate and clinical outcomes of symptomatic VTE among Asian patients with advanced pancreatic cancer.

In our study, 8.0% (99.0 per 1000 person-years) of patients with pancreatic cancer developed VTE after chemotherapy initiation. A higher incidence of VTE among patients with pancreatic cancer was found in our study than in a national study in Taiwan conducted between 2001 and 2008, in which the incidence rate of VTE was 3.1% (27.8 per 1000 person-years) among patients with newly diagnosed pancreatic cancer who required hospitalization for VTE treatment [10]. The discrepancy in the results may be explained by the advanced tumor stage of our study group (all patients had stage III or IV), distinct severity of VTE episode, more awareness of VTEs, and wide application of imaging studies in the modern era. Recently, Kondo et al. reported the incidence rate of VTE was 16.5% (17 of 103 patients) in previously untreated pancreatic cancer patients from Japanese cohort. However, the majority of patients with VTE were asymptomatic because all patients were screened for VTE at the time of cancer diagnosis [26]. Yoon et al. reported that the VTE occurred in 18.6% (94 of 505 patients) of Korean patients with advanced pancreatic cancer; however, 7.5% of the patients had isolated splanchnic vein thrombosis, which were incidentally diagnosed by image study [27]. An early study reported that 5.3% of Korean pancreatic cancer patients developed VTE after diagnosis of pancreatic cancer [23]. Recently, Lee et al. reported that the 2-year cumulative incidence of symptomatic VTE was 9.2% by studying 1,115 Korean pancreatic cancer patients; VTE incidence in their study was even lower than that observed in the current study (2-year cumulative incidence rate: 19.4%) as they included patients irrespective of pancreatic cancer stage [28]. Nevertheless, VTE incidence in Asian patients with pancreatic cancer is significantly lower than that in the Western counterparts. One nationwide study in the United States showed that VTE occurred in 12.6% of 17,284 patients with various cancer types between 2004 and 2009, and patients with pancreatic cancer had the highest incidence of VTE (19.2%) [29]. A retrospective study conducted at Memorial Sloan-Kettering Cancer Center (MSKCC) reported that up to 36% of 1915 patients with pancreatic cancer had VTE following chemotherapy [30]. Using California Cancer Registry data, Chew et al. reported the incidence rate of VTE was 200 per 1000 person-years among patients with metastatic pancreatic cancer [12]. Therefore, the incidence of VTE in our study group was half of that in Western patients with advanced pancreatic cancer.

In our study, presence of liver metastases and leukocytosis were associated with increased risks of VTE. These results are consistent with those of previous studies in patients with cancer [24,31]. The liver is one of the most common metastatic sites of pancreatic cancer. Presence of liver metastases indicates a metastatic tumor stage and possibly a huge tumor burden, which has been confirmed by several studies that have suggested that patients with advanced tumor stages have a higher risk of VTE than those with early tumor stages [7,11,12,25]. However, none of these studies have specifically analyzed the risk of VTE among patients with metastases in different organs. The association between liver metastases and VTE that was observed in our study must be confirmed by conducting additional large-scale clinical studies.

Khorana et al. developed a risk scoring system (Khorana risk score) to predict symptomatic VTE occurrence in patients with cancer receiving chemotherapy; the scoring system consists of five variables, including cancer site; platelet count ≥ 350 × 10^9^/L, hemoglobin < 10 g/dL, and/or erythropoietin and leukocyte counts > 11,000/μL; and BMI ≥ 35 kg/m^2^ [24]. The higher rate of symptomatic VTE occurrence was externally validated in Western patients possessing more risk factors [32]. In our study, patients presenting with higher Khorana risk scores tended to develop VTE; however, of the four variables of the Khorana risk scoring system (excluding tumor site), only the leukocyte count was a significant predictor of VTE in our study group. Patients’ characteristics and anticancer treatments for pancreatic cancer were different between Western and Asian populations. For example, in our study group, none of the patients presented with BMI ≥ 35 kg/m^2^ or received erythropoietin treatment. The incidence rate of VTE is low in Asian patients with pancreatic cancer, furthermore, it is not suitable to use Khorana risk score to predict VTE in pancreatic cancer patients because pancreatic cancer itself is a one of the criteria of this points-scoring system. Our result showed that the clinical application of the Khorana risk score is limited in Asian patients with pancreatic cancer.

Sorenson et al. first reported that cancer patients with VTE had poorer survival outcomes than those without VTE [15]. Subsequent reports have supported the finding that VTE is a negative prognosticator in patients with cancer [12,14,33]. However, some recent studies have reported that VTE has no influence on survival in patients with breast cancer [34], gastric cancer [35], or pancreatic cancer [21,28]. For example, Lee et al. reported that advanced cancer stage was the most important factor affecting VTE occurrence and mortality, while VTE occurrence did not affect survival in pancreatic cancer patients [28]. In contrast, the retrospective study conducted at MSKCC showed that pancreatic cancer patients with early thrombosis (defined as thrombosis occurrence within 1.5 months after chemotherapy initiation) exhibited a significantly increased risk of death than those with late thrombosis or no thrombosis [30]. Similarly, our study found that only early-onset VTE, but not all VTE or late-onset VTE events, was associated with poor survival outcomes in patients with advanced pancreatic cancer following chemotherapy. Patients with VTE are more likely to exhibit advanced tumor stages and comorbidities, which might directly affect survival outcomes [6,7,11,12,36,37]. Therefore, the true effect of VTE on survival outcome after adjusting for tumor types, stages, comorbidities, duration of VTE occurrence after cancer diagnosis, and other confounding factors must be further studied and explored.

In this study, half of our patients developed VTE within 4 months following chemotherapy. Studies have reported that the incidence of VTE was the highest in the period immediately following diagnosis or anticancer treatment, and the incidence declined rapidly to a constant level over time [9,14,38]. Several factors might contribute to this finding. First, the initiation of aggressive antitumor treatments may increase the risk of VTE. For example, surgical resection and cytotoxic chemotherapy are associated with an increased risk of VTE [26,29,37,39,40,41,42]. Second, the increasing application of indwelling catheters in upper extremities as vascular access for chemotherapy delivery before initiating anticancer treatment might lead to a predisposition toward upper extremity VTE [43]. Third, the common use of some adjuvant agents in aggressive treatment, such as erythropoietin-stimulating agent, myeloid growth factor, or megestrol acetate, might increase the risk of VTE [29,36,38,44,45]. Finally, a high proportion of patients with newly diagnosed cancer or those beginning antitumor treatment might be required to be hospitalized with prolonged bed rest or immobilization, which are associated with an increased risk of VTE [6].

This is the first study to evaluate the incidence of VTE, its risk factors, and its effect on the survival outcomes of Asian patients with pancreatic cancer receiving palliative chemotherapy. The strength of our study is that a large number of patients were enrolled from multiple centers across Taiwan and were evaluated over a 7-year period. However, our study has some limitations. First, as an inherent limitation of the retrospective study design, selection bias might exist. Second, in the chart review, it was difficult to classify whether the main cause of death was VTE or cancer. Third, the incidence of VTE might be underestimated in our study group, as some patients were too ill to receive examination for confirmation of VTE. Finally, Maraveyas et al. randomized 123 advanced pancreatic cancer patients to receive either gemcitabine or gemcitabine plus dalteparin for 12 weeks [19]. VTE incidence reduced from 28% to 12% in the whole follow-up period, with a 58% risk reduction. Similarly, Pelzer et al. reported a 60% risk reduction of symptomatic VTE among 312 advanced pancreatic cancer patients receiving prophylactic enoxaparin and chemotherapy or chemotherapy alone [20]. Most importantly, the number of major bleeding events was similar between the two groups. Because none of the patients received thromboprophylaxis for VTE prevention in our patient group, we were unable to evaluate the efficacy and safety profile of thromboprophylaxis in our Asian pancreatic cancer patients. A future prospective study should address these limitations.

## 4. Patients and Methods

### 4.1. Patient Selection

To determine VTE incidence after chemotherapy initiation, the medical records of inpatients with newly diagnosed locally advanced or metastatic pancreatic cancer who underwent palliative chemotherapy from 2010 to 2016 at four institutes; Linkou, Keelung, Chiayi, and Kaohsiung branches of Chang Gung Memorial Hospital (CGMH); in Taiwan were retrospectively reviewed. All patients were either pathologically or radiographically diagnosed with primary pancreatic cancer and received palliative chemotherapy for the treatment of pancreatic cancer. In patients in whom histological confirmation was not possible, imaging study results of either computed tomography or magnetic resonance cholangiopancreatography as well as abnormal carcinoembryonic antigen (CEA) or carbohydrate cell surface antigen19-9 (CA19-9) levels are mandatory for pancreatic cancer diagnosis. Patients who had recurrent tumor after radical surgery or concurrent other active malignancy were excluded. Finally, a total of 838 consecutive patients were enrolled into this study. Patients were categorized into non-VTE and VTE groups based on whether they had VTE after the initiation of palliative chemotherapy. VTE was diagnosed by the treating clinician based on clinical symptoms and results of imaging studies, including venous duplex imaging, computed tomography, and magnetic resonance imaging. Arterial thrombotic events were not included in the analysis. Patients with VTE occurrence within and after the first 1.5 months were included in the early-onset and late-onset VTE groups, respectively [30]. The incidence and clinical characteristics of the 838 patients were analyzed to identify independent predictors of VTE and its effect on the survival outcome. This study was approved by the institutional review boards of all the CGMH branches at 22 November 2017 (ethic code: 201701796B0) and has been conducted in compliance with the Helsinki Declaration (1996).

### 4.2. Data Collection

Patients’ demographic and clinical data, including age, sex, BMI, Eastern Cooperative Oncology Group performance status (ECOG PS), pre-existing comorbidities evaluated using the modified CCI, anatomic location of the primary cancer, clinical stage, presence of drainage for obstructive jaundice, serum CEA and (CA19-9) levels, parameters of complete peripheral blood count, metastatic site, and chemotherapy regimens, were recorded by the primary care physician by using a prospectively formulated electronic data form obtained from our previous studies [46,47]. The same cutoff value reported for the Khorana risk score was used for the complete blood count in our study. All included patients were followed up until death or 31 December 2017. OS and cumulative incidence of VTE were calculated from the date of chemotherapy initiation to the date of death from any cause and VTE occurrence, respectively. All dates of death were obtained from either the Institutional Cancer Registry or the National Registry of Death database in Taiwan.

### 4.3. Statistical Analysis

Basic demographic data are summarized as n (%) for categorical variables and as median with range, standard error, or 95% confidence interval (CI) for continuous variables. Differences between non-VTE and VTE groups were determined using the Pearson χ^2^ test or Fisher’s exact test if the number of variables in any cell was less than five. Univariate and multivariate analyses of all clinical factors for determining OS were performed using the log-rank test and Cox’s proportional hazard model. Moreover, univariate and multivariate logistic regression analyses were performed to investigate risk factors for VTE. All variables in univariate analysis with *p* values < 0.20 were further analyzed using multivariate analysis. Statistical analysis was conducted using SPSS 17.0 software (SPSS Inc., Chicago, IL, USA). All statistical assessments were two-sided, and a *p* value of < 0.05 was considered statistically significant.

## 5. Conclusions

The incidence of VTE was found to be lower in Asian patients with pancreatic cancer receiving palliative chemotherapy than in their Western counterparts. The Khorana risk score was not a predictor of VTE in the Asian patients. Only patients with early-onset VTE, but not late-onset VTE, had a poorer prognosis than those without VTE. Awareness of the incidence, clinical characteristics, and survival outcomes of Asian pancreatic cancer patients with VTE may assist clinicians and patients in choosing the appropriate prophylaxis and management strategy for VTE.

## Figures and Tables

**Table 1:** Patients’ demographic data.

Variable	Category	All Patients, *n* = 838 (%)	Patients Without VTE, *n* = 771 (%)	VTE Patients, *n* = 67 (%)	*p*
Age, year	median (range)	62 (23–89)	62 (23–89)	63(43–83)	0.86
Sex					0.52
	male	497(59.3)	460 (59.6)	37 (56.1)	
	female	341 (40.7)	311 (40.3)	30 (44.8)	
BMI, kg/m^2^	median (range)	22.5 (13–36.2)	22.4 (13.0–36.0)	22.8 (15.9–36.2)	0.25
BMI, kg/m^2^	>35	3 (0.4)	2 (0.3)	1 (1.5)	0.22
ECOG PS					0.48
	0–1	597 (71.2)	545 (70.7)	52 (77.6)	
	2	206 (24.6)	193 (25.0)	13 (19.4)	
	3	35 (4.2)	33 (4.3)	2 (3.0)	
CCI					0.79
	0	227 (27.1)	211 (27.4)	16 (23.9)	
	1	292 (34.8)	265 (34.4)	27 (40.3)	
	2	193 (23.0)	179 (23.2)	14 (20.9)	
	>3	126(15.0)	116 (15.0)	10 (14.9)	
Diabetes mellitus	presence	313 (37.4)	292 (37.9)	21 (31.3)	0.37
Hypertension	presence	332 (39.6)	302 (39.2)	30 (44.8)	0.36
Cerebrovascular disease	presence	30 (3.6)	28 (3.6)	2 (3.0)	0.99
Coronary artery disease	presence	52 (6.2)	46 (6.0)	6 (9.0)	0.29
Arrhythmia	presence	13 (1.6)	12 (1.6)	1 (1.5)	0.99
Tumor site					0.12
	head	343 (40.9)	320 (41.5)	23 (34.3)	
	body	148 (17.7)	140 (18.2)	8 (11.9)	
	tail	171 (20.4)	156 (20.2)	15 (22.4)	
	overlapping	176 (21.0)	155 (20.1)	21 (31.3)	
Tumor stage, seventh AJCC					0.35
	III	183 (21.8)	172 (22.3)	11 (16.4)	
	IV	655 (78.2)	599 (77.7)	56 (83.6)	
Tumor grade					0.53
	well to moderate differentiation	93 (11.1)	83 (10.8)	10 (14.9)	
	poorly differentiation	92 (11.0)	86 (11.2)	6 (9)	
	unclassified or unknown	653 (77.9)	602 (78.1)	51 (76.1)	
Jaundice under drainage	presence	272 (32.5)	248 (32.2)	24 (32.2)	0.59
Metastatic organ	liver	438 (52.3)	394 (51.1)	44 (65.7)	0.03
	peritoneum	239 (28.5)	222 (28.8)	17 (25.4)	0.67
	lymph nodes	150 (17.9)	138 (17.9)	12 (17.9)	0.99
	lung	98 (11.7)	88 (11.4)	10 (14.9)	0.43
CEA, ng/mL	>5	427 (51.0)	387 (50.2)	40 (59.7)	0.16
CA19-9, u/mL	>37	666 (79.5)	610 (79.1)	56 (83.6)	0.43
Platelet count, 10^9^/L	>350	86 (10.3)	81 (10.5)	5 (7.5)	0.53
Hemoglobin, g/dL	>10	734 (87.6)	674 (87.4)	60 (89.6)	0.70
Leukocyte count, 10^9^/L	>11	167 (19.9)	146 (18.9)	21 (31.3)	0.024
Chemotherapy regimen had been exposure	Gemcitabine	792 (94.5)	726 (94.2)	66 (98.5)	0.17
	5-FU or S-1	592 (70.6)	540 (70.0)	52 (77.6)	0.45
	Platinum	433 (51.7)	401 (51.9)	32 (48.5)	0.61
Khorana risk score					0.33
	2	60 (7.2)	56 (7.3)	4 (6.0)	
	3	591 (70.5)	549 (71.2)	42 (62.7)	
	4	162 (19.3)	144 (18.7)	18 (26.9)	
	5	25 (3.0)	22 (2.9)	3 (4.5)	

BMI, body mass index; ECOG PS, Eastern Cooperative Oncology Group performance status; CCI, Charlson comorbidity index; AJCC, American Joint Committee on Cancer; CEA, carcinoembryonic antigen, CA19-9, Carbohydrate antigen19-9; 5-FU, 5-fluorouracil.

**Table 2:** Distribution of venous thromboembolism in our study group (*n* = 67).

Location of thrombosis	*n*	%
DVT, lower limb	34	50.7%
DVT, upper limb	7	10.4%
PE	14	20.9%
Concomitant DVT and PE	8	11.9%
Concomitant DVT and IVC thrombosis	2	3.0%
Concomitant upper and lower limb DVT	1	1.5%
Concomitant lower limb DVT and PVT	1	1.5%

DVT, deep vein thrombosis; PE, pulmonary embolism; PVT, portal vein thrombosis; IVC; inferior vena cava.

**Table 3:** Univariate and multivariate analyses of clinical factors for predicting venous thromboembolism occurrence.

Clinical Factors	Univariate Analysis				Multivariate Analysis		
	HR	SE	95% CI	*p*	HR	95% CI	*p*
Clinical characteristics							
Male (vs. female)	1.20	0.25	0.73–1.98	0.48			
Age > 65 years (vs. ≤ 65 years)	0.83	0.26	0.50–1.39	0.49			
Presence of hypertension (vs. no hypertension)	1.26	0.26	0.76–2.08	0.37			
Presence of diabetes mellitus (vs. no diabetes mellitus)	0.75	0.27	0.44–1.28	0.29			
Presence of coronary artery disease (vs. no coronary artery disease)	1.55	0.45	0.64–3.78	0.33			
Presence of cerebrovascular disease (vs. no cerebrovascular disease)	0.82	0.74	0.19–3.50	0.79			
ECOG PS 0–1 (vs. > 1)	0.70	0.30	0.38–1.26	0.23			
CCI 0 (vs. > 0)	1.20	0.30	0.67–2.15	0.54			
BMI > 23 kg/m^2^ (≤23 kg/m^2^)	1.06	0.26	0.64–1.75	0.82			
Tumor characteristics							
Tumor site at pancreatic head (vs. other sites)	0.74	0.27	0.44–1.24	0.25			
Presence of jaundice under drainage (vs. no jaundice)	1.18	0.27	0.70–1.98	0.54			
Stage IV disease (vs. stage III)	1.46	0.34	0.75–2.85	0.27			
Presence of liver metastases (vs. no liver metastases)	1.83	0.27	1.08–3.09	0.024	1.65	1.03–3.99	0.046
Presence of peritoneal metastases (vs. no peritoneal metastases)	0.84	0.29	0.48–1.49	0.55			
Presence of lung metastases (vs. no lung metastases)	1.36	0.36	0.67–2.76	0.39			
Presence of distant lymph nodes metastases (vs. no distant lymph node metastases)	1.01	0.33	0.52–1.92	0.99			
Biological data							
CEA > 5 ng/mL (vs. < 5 ng/mL)	1.47	0.26	0.88–2.44	0.14	1.27	0.75–2.14	0.37
CA19-9 > 37 u/mL (≤37 u/mL)	1.34	0.34	0.69–2.62	0.39			
Platelet count > 350 × 10^9^/L (vs. ≤ 350 × 10^9^/L)	0.69	0.48	0.27–1.76	0.43			
Hemoglobin > 10 g/dL (vs. ≤ 10 g/dL)	1.23	0.41	0.55–2.78	0.61			
Leukocyte count > 11,000/μL (vs. ≤ 11,000/μL)	1.95	0.28	1.13–3.38	0.016	1.75	1.07–3.03	0.032
Use of anticancer drugs							
Chemotherapy with platinum (vs. without platinum)	0.90	0.26	0.55–1.48	0.68			
Chemotherapy with gemcitabine (vs. without gemcitabine)	4.09	1.02	0.56–30.2	0.17	4.27	0.58–21.0	0.16
Chemotherapy with 5-FU or S-1 (vs. without 5-FU or S-1)	1.21	0.27	0.72–2.03	0.48			
Khorana risk score 4–5 (vs. score 2–3)	1.66	0.28	0.97–2.87	0.067	1.12	0.36–2.64	0.66

ECOG PS, Eastern Cooperative Oncology Group performance status; CCI, Charlson comorbidity index; BMI, body mass index; CEA, carcinoembryonic antigen, CA19-9, Carbohydrate cell surface antigen19-9; 5-FU, 5-fluorouracil; HR, hazard ratio, SE, standard error; CI, confidence interval.

**Table 4:** Venous thromboembolism incidence rate and hazard ratios (HR) among patients with pancreatic cancer, stratified by Khorana risk score.

Khorana Risk Score	Overall, *n* = 838	2, *n* = 60	3, *n* = 591	4, *n* = 162	5, *n* = 25
Venous thromboembolism, *n*	67	4	42	18	3
Person-years	676.9	39.2	510.7	113.8	13.2
Incidence rate, per 1000 person-years	99.0	102.0	82.2	158.2	227.3
Crude HR (95% CI)		1	0.90 (0.32–2.50)	1.73 (0.59–5.12)	2.24 (0.50–10.0)
Adjusted HR* (95% CI)		1	0.97 (0.35–2.70)	1.20 (0.31–4.67)	1.23(0.21–7.19)

* The HR was adjusted for Leukocyte count and liver metastases.

**Table 5:** Univariate and multivariate analyses of clinical factors for determining overall survival.

Clinical Factor	Univariate Analysis				Multivariate Analysis		
	HR	SE	95% CI	*p*	HR	95% CI	*p*
Clinical characteristics							
Male (vs. female)	1.31	0.08	1.13–1.52	<0.001	1.27	1.09–1.47	0.002
Age > 65 years (vs. age ≤ 65)	1.21	0.07	1.05–1.40	0.011	1.07	0.91–1.25	0.41
ECOG PS 0–1 (vs. ECOG PS >1)	0.37	0.08	0.31–0.43	<0.001	1.78	1.48–2.14	<0.001
CCI 0 (vs. CCI > 0)	0.66	0.08	0.56–0.78	<0.001	1.37	1.15–1.63	<0.001
BMI > 23 kg/m^2^ (vs. ≤ 23 kg/m^2^)	0.93	0.07	0.81–1.08	0.33			
Tumor characteristics							
Tumor site at pancreatic head (vs. other sites)	1.07	0.07	0.93–1.24	0.35			
Presence of jaundice under drainage (vs. no jaundice)	1.11	0.08	0.95–1.29	0.21			
Stage IV disease (vs. stage III)	1.99	0.09	1.66–2.40	<0.001	2.04	1.69–2.46	<0.001
Biological data							
CEA > 5 ng/mL (vs. ≤ 5 ng/mL)	1.28	0.07	1.11–1.48	0.001	1.10	0.95–1.28	0.21
CA19-9 > 37 u/mL (vs. ≤ 37 u/mL)	1.06	0.09	0.88–1.26	0.56			
Platelet count > 350 × 10^9^/L (vs. ≤ 350 × 10^9^/L)	1.09	0.09	0.87–1.40	0.44			
Hemoglobin > 10 g/dL (vs. ≤ 10 g/dL)	0.64	0.11	0.51–0.79	<0.001	1.15	0.92–1.45	0.22
Leukocyte count > 11,000/μL (vs. ≤ 11,000/μL)	1.69	0.09	1.41–2.01	<0.001	1.24	1.03–1.49	0.02
Use of anticancer drugs							
Chemotherapy with platinum (vs. without platinum)	0.62	0.07	0.53–0.71	<0.001	1.42	1.21–1.66	<0.001
Chemotherapy with gemcitabine (vs. without gemcitabine)	0.50	0.16	0.37–0.68	<0.001	2.22	1.59–3.10	<0.001
Chemotherapy with 5-FU or S-1 (vs. without 5-FU or S-1)	0.44	0.08	0.38–0.51	<0.001	1.93	1.64–2.27	<0.001
Occurrence of VTE							
Occurrence of any VTE (vs. no VTE)	1.15	0.13	0.89–1.50	0.29			
Early onset of venous thromboembolism (vs. no VTE)	4.86	0.24	3.02–7.80	<0.001	3.80	2.34–6.18	<0.001
Late onset of venous thrombosis (vs. no VTE)	0.88	0.16	0.64–1.91	0.39			

ECOG PS, Eastern Cooperative Oncology Group performance status; CCI, Charlson comorbidity index; BMI, body mass index; CEA, carcinoembryonic antigen, CA19-9, Carbohydrate cell surface antigen19-9; 5-FU, 5-fluorouracil; VTE, venous thromboembolism; HR, hazard ratio, SE, standard error; CI, confidence interval.
